# Role of heparan sulfate in mediating CXCL8-induced endothelial cell migration

**DOI:** 10.7717/peerj.1669

**Published:** 2016-02-04

**Authors:** Zhiping Yan, Jingxia Liu, Linshen Xie, Xiaoheng Liu, Ye Zeng

**Affiliations:** 1 Institute of Biomedical Engineering, School of Preclinical and Forensic Medicine, Sichuan University, China; 2 West China School of Public Health, No. 4 West China Hospital, Sichuan University, China

**Keywords:** Heparan sulfate, CXCL8, HUVEC, Rho GTPases, Cell migration

## Abstract

CXCL8 (Interleukin-8, IL-8) plays an important role in angiogenesis and wound healing by prompting endothelial cell migration. It has been suggested that heparan sulfate (HS) could provide binding sites on endothelial cells to retain and activate highly diffusible cytokines and inflammatory chemokines. In the present study, we aimed to test the hypothesis that HS is essential for enhancement of endothelial cell migration by CXCL8, and to explore the underlying mechanism by detecting the changes in expression and activity of Rho GTPases and in the organization of actin cytoskeleton after enzymatic removal of HS on human umbilical vein endothelial cells (HUVECs) by using heparinase III. Our results revealed that the wound healing induced by CXCL8 was greatly attenuated by removal of HS. The CXCL8-upregulated Rho GTPases including Cdc42, Rac1, and RhoA, and CXCL8-increased Rac1/Rho activity were suppressed by removal of HS. The polymerization and polarization of actin cytoskeleton, and the increasing of stress fibers induced by CXCL8 were also abolished by heparinase III. Taken together, our results demonstrated an essential role of HS in mediating CXCL8-induced endothelial cell migration, and highlighted the biological importance of the interaction between CXCL8 and heparan sulfate in wound healing.

## Introduction

Inflammatory chemokines has been shown to rapidly accumulate in vascular injury. CXCL8 are among the inflammatory chemokines known to upregulate endothelial cell (EC) adhesion molecules, to recruit leukocytes and to direct EC and smooth muscle cell migration, contributing to the wound healing in vascular homeostasis and overall health. CXCR1 and CXCR2 are two kinds of G protein-coupled receptors for CXCL8 (Zeng et al., 2011). Activation of those receptors by CXCL8 causes phosphorylation of protein kinase B, calcium influx, formation of F-actin, and cytoskeleton rearrangement. Those events are very important for directed chemotactic movement of leukocytes and ECs (Baggiolini, 2015; Gales et al., 2013). It was recently demonstrated that CXCL8 could provide binding sites for the negatively-charged endothelial glycosaminoglycan (GAGs) in endothelial cells (Pichert et al., 2012a). Those binding sites for GAGs are well separated with the binding sites for CXCL8 receptors, allowing CXCL8 to interact closely with both components simultaneously (Pichert et al., 2012b; Tarbell, Simon & Curry, 2014). GAGs binding to CXCL8 could promote the oligomerization and retention of CXCL8 on cell surface (Proudfoot, 2006), which can build up a chemotactic gradient at inflammatory loci and thereby cause a priming of cells and a modulation of the cell migration spatiotemporally. The immobilization of CXCL8 on the glycocalyx on the abluminal surface of the endothelium modulates the leukocyte recruitment (Carveth et al., 1989). Once the C-terminal GAG-binding domain on CXCL8 was deleted, it failed to attract leukocyte (Middleton et al., 1997), indicating that the binding of CXCL8 to surface endothelial GAGs is a crucial prerequisite for cell motility.

GAGs are heterogeneous unbranched polysaccharides with high charge densities (Fu & Tarbell, 2013; Tarbell & Ebong, 2008; Tarbell & Pahakis, 2006; Weinbaum, Tarbell & Damiano, 2007). The most prominent GAG in the vasculature is heparan sulfate (HS), accounts for >50% of the total GAG, usually is present in the endothelial glycocalyx layer as proteoglycan attachment (Tarbell, Simon & Curry, 2014). The recent work has clearly demonstrated the importance of HS in the EC motility (Moon et al., 2005; Thi et al., 2004; Yao, Rabodzey & Dewey, 2007). However, the effect and its underlying mechanism of HS in CXCL8-induced EC migration are still unclear.

Cell migration was mechanistically regulated by intertwined signaling networks (Vicente-Manzanares, Webb & Horwitz, 2005). Rho-family GTPases (Rho GTPases, including Cdc42, Rac1, and RhoA) play central roles in cell migration (Nobes & Hall, 1995). For example, Cdc42 controls the formation of protrusion and filopodia. Rac1 induces the formation of protrusion and lamellipodia. RhoA mediates the stress fibers formation. Our previous work has demonstrated that CXCL8 induces the endothelial cell (EA.hy926 cell line) migration via Rac1/RhoA pathway (Lai et al., 2011).

In the present study, we detected the roles of HS in CXCL8-induced HUVEC migration, and its roles in regulating Rho GTPases and actin cytoskeleton by CXCL8. Our results demonstrated an essential role of HS in mediating CXCL8-induced endothelial cell migration, and highlighted the biological importance of the interaction between CXCL8 and heparan sulfate in wound healing.

## Material and Methods

### Cell culture

Human umbilical vein endothelial cells (HUVECs) were purchased from Allcells, China. HUVECs were grown in complete culture media for HUVECs (Allcells, China) in a humidified 5%/95% CO_2_/air incubator at 37 °C. Fluorescence-activated cell sorting (FACS) analysis showed that almost all of the cells (>96%) take up high amount of acetylated LDL and are positive for the presence of CD31, CD45, CD62, and von Willebrand factor (vWF). Cells (passages 3–5) were plated on to glass slides or 6-well plates at a density of 1 × 10^5^ cells/cm^2^ and cultured for 3–5 days until they attained confluence.

### Heparinase III and/or CXCL8 treatments

F. heparinum heparinase III (Aglyco, Beijing, China) selectively cleaves HS of the glycocalyx (Zeng et al., 2012a). It was demonstrated that HS was dramatically degraded by 15 mU/ml heparinase III in ECs, including HUVECs (Giantsos-Adams et al., 2013). Cells were treated with 15 mU/ml heparinase III and/or 100 ng/ml CXCL8 (Pepro-Tech, NJ, USA) in basic HUVEC culture medium (Allcells, Shanghai, China) with 1% bovine serum albumin (BSA) for indicated times, respectively.

### Scratch wound assay

For scratch wound assay (Zeng et al., 2011), prior to the heparinase III/CXCL8 treatments, HUVEC monolayers were scratched by a yellow tip after confluence in 6-well plates, followed by twice-washed to remove the cell debris. After treatment for indicated times, photographs were taken with an invert contrast microscopy (Olympus CKX41, Jap) and digitized using a digital camera (Cannon Powershot G11), and the wound areas were calculated to evaluate the cell migration capacity by using ImageJ 1.50b Gel Analyzer (National Institutes of Health, Washington, D.C., USA).

### Western blot

After treatments, expressions of Rho GTPases in HUVECs were performed by Western Blot. Cells were washed and then lysed on ice for 10 min in RIPA Lysis Buffer (Beyotime, Jiangsu, China) with an addition of protease inhibitor cocktail (1:100, BestBio Science, Shanghai, China), phosphatase inhibitor cocktail (1:100; BestBio Science, Shanghai, China) and 10 mM phenylmethylsulfonyl fluoride (PMSF). Protein concentration was measured by a Protein Determination Kit (Cayman Chemical, Ann Arbor, MI, USA). Proteins were size fractionated using SDS-PAGE and electrotransferred onto PVDF membrane (Bio-Rad, Hercules, CA, USA), and hybridized with monoclonal antibodies (Santa Cruze, USA) including mouse anti-RhoA antibody (1:500), Rabbit anti-Rac1 antibody (1:200) and mouse anti-Cdc42 antibody (1:200). Detection was carried out using peroxidase-conjugated secondary antibodies (goat anti-mouse or goat anti-rabbit, 1:5,000) and enhanced chemiluminescence reagents (BeyoECL Plus, Beyotime, Jiangsu, China). Blots were imaged by Molecular Image^®^ ChemiDoc™ XRS+ with Image Lab™ Software (Bio-Rad, Hercules, CA, USA). Quantitative data were obtained by using ImageJ 1.50b Gel Analyzer.

### Rho GTPases activity assay

Cells after treatment were lysed and 500 µg protein was used in a pulldown assay with GST-human Pak1-PBD (for Rac1) or GST-Rhotekin-RBD (for RhoA) beads, and the resulting pulldown was then immunoblotted using an active Rac1/Rho pulldown and detection kit (Thermo Scientific, Waltham, MA, USA) according to the manufacturer’s instructions.

### Visualization of actin cytoskeleton and confocal microscopy

Immediately after treatments, HUVECs were fixed in 2% paraformaldehyde, permeabilized with 1% Triton X-100, and stained with BODIPY^®^ FL phallacidin (Invitrogen, USA) to visualize the actin cytoskeleton. Cell nuclei were stained by DAPI. All samples were imaged with a Leica TCS SP5 laser scanning confocal microscopy (Sichuan University). The max-intensity Z-projection images were shown as described previously (Zeng et al., 2014; Zeng et al., 2012a; Zeng & Tarbell, 2014; Zeng et al., 2013).

### Quantitation of subcellular actin pattern

Quantitative approaches were used to analysis the subcellular actin pattern. For stress fibers, the thickness and relaxation rate (defined as ratio of actual length of a stress fiber to shortest distance between both ends) were obtained by using ImageJ. For stress bands, we measured the bands width.

### Statistical analysis

Data are presented as means ± SD. Statistical analysis was performed by one-way ANOVO with either the least significant difference (LSD) test or Tamhane’s T2 test (depending on Levene’s statistic for homogeneity of variance), using the SPSS 21 software package. Differences in means were considered significant if *P* < 0.05.

## Results

### An essential role of heparan sulfate in CXCL8-induced HUVEC migration

**Figure 1:**
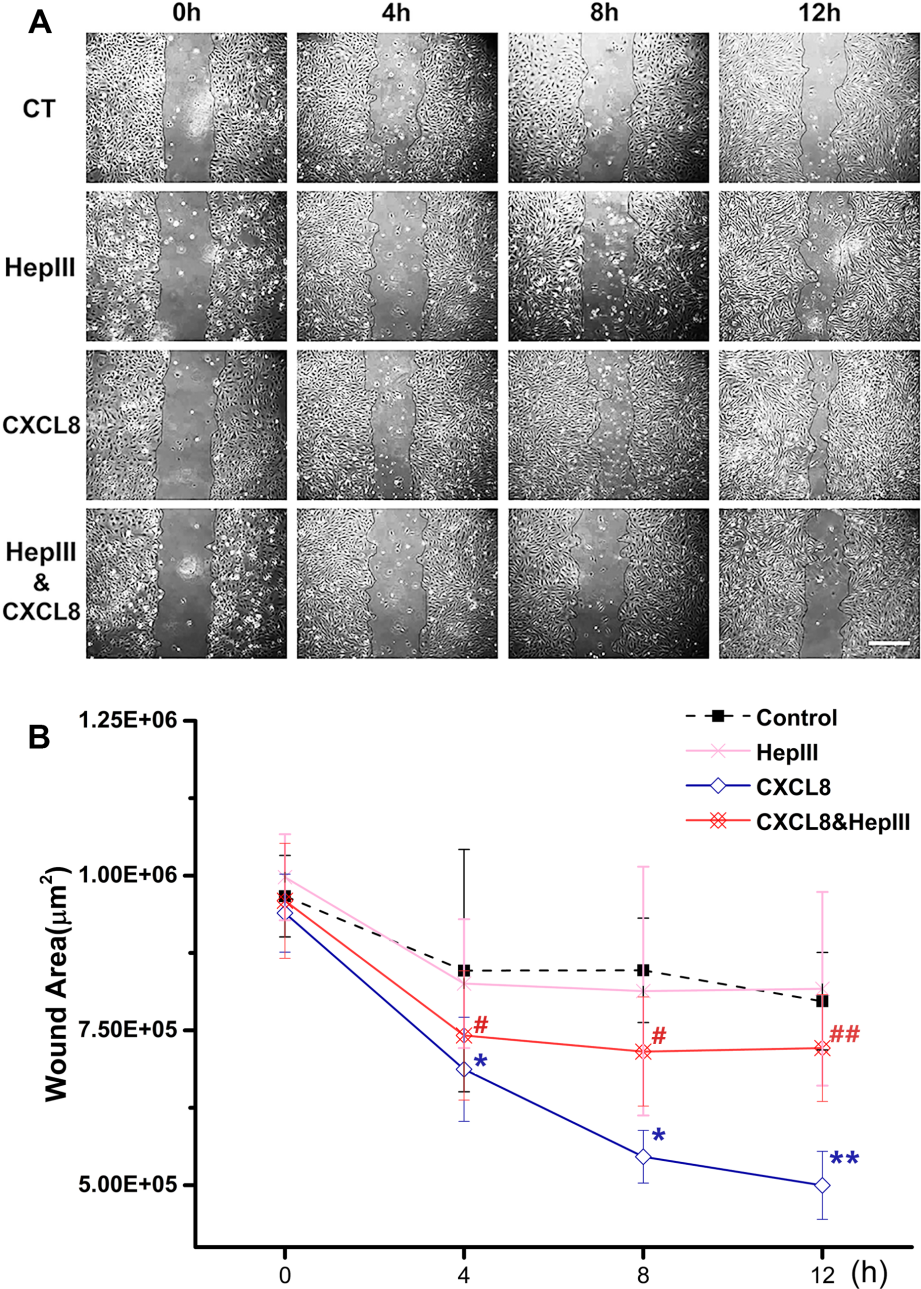
Heparinase III attenuated the CXCL8-induced wound healing. HUVEC monolayers were scratched by a yellow tip after confluence in 6-well plates, followed by twice-washed to remove the cell debris, and then were treated with 15 mU/ml heparinase III (HepIII), 100 ng/ml CXCL8, or both for indicated times (4, 8, and 12 h), respectively. Normal cell without treatment was set as control (CT). At each time point, photographs were taken (A), and the wound areas were calculated to evaluate the cell migration capacity (B). CXCL8 induced wound healing suggesting CXCL8 promotes cell migration, and this could be attenuated by heparinase III. Scale bar: 400 µm; *n* = 4; ^∗^*P* < 0.05, ^∗∗^*P* < 0.01 vs. control; #*P* < 0.05 vs. CXCL8 at each time point.

The migration capacity of HUVEC was evaluated by wound assay (Fig. 1). The wound area was decreased gradually with the CXCL8 treatment time. There was significant difference between the CXCL8-treated group and controls after 4 h, suggesting that CXCL8 induced the healing of wound area and promoted HUVEC migration. By treated with heparinase III and CXCL8 concomitantly, the healing of wound area was greatly attenuated. When treated with heparinase III alone, the wound area was not significantly changed compared with control. Taken together, HS plays an essential role in the CXCL8-induced HUVEC migration.

### Heparan sulfate mediates the CXCL8-regulated Cdc42 expression in HUVEC

**Figure 2:**
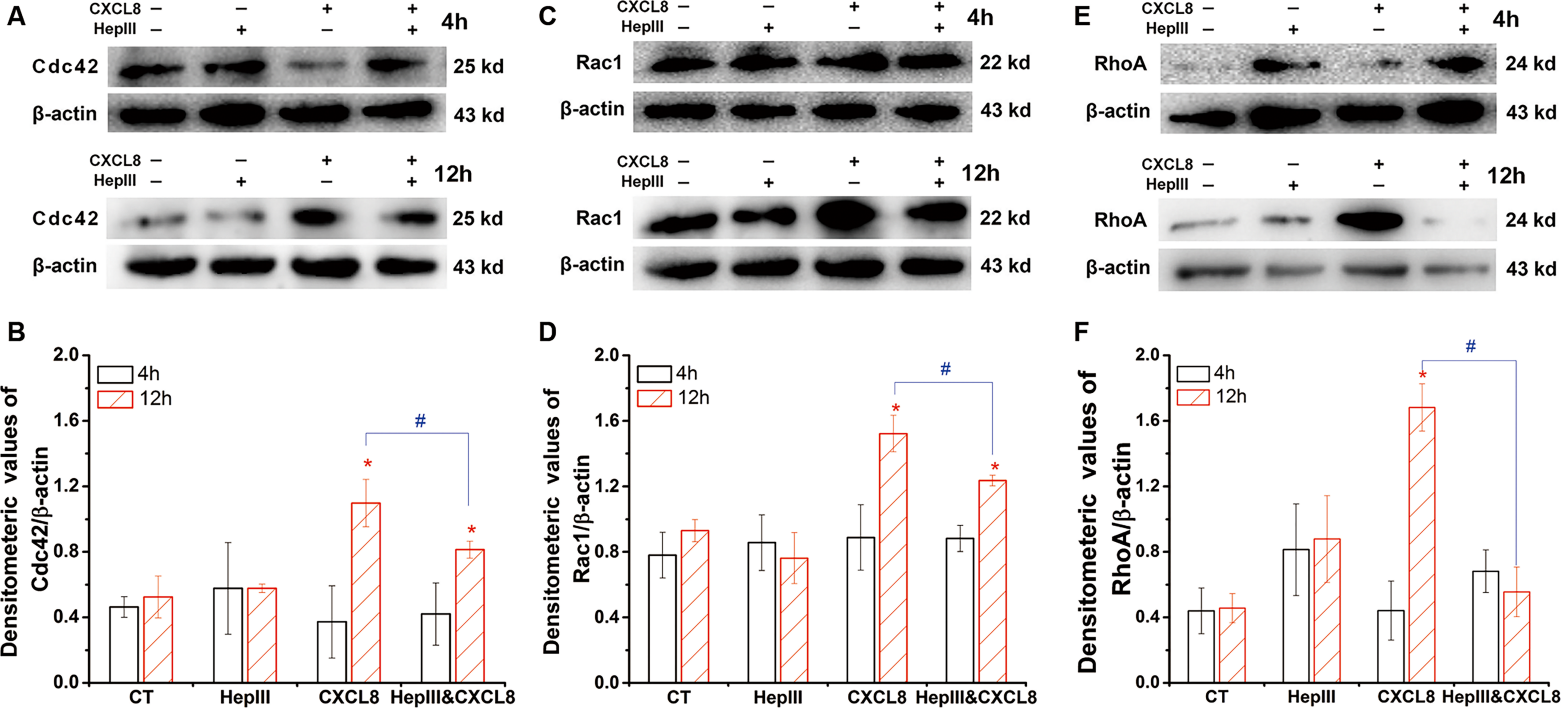
Effect of heparinase III on the CXCL8-modulated expression of Rho GTPases. HUVECs were treated with 15 mU/ml heparinase III (HepIII), 100 ng/ml CXCL8, or both for indicated times (4 and 12 h), respectively. Normal cell without treatment was set as control (CT). The expression of Rho-GTPases was detected by western blot, and quantitative data were obtained using ImageJ: (A and B) Cdc42; (C and D) Rac1; (E and F) RhoA. ^∗^*P* < 0.05 vs. CT at 12 h; #*P* < 0.05.

The expression of Cdc42 induced by CXCL8 was time-dependent (Figs. 2A and 2B). At 4 h, Cdc42 was slightly upregulated by heparinase III with or without CXCL8 (Fig. 2A), with no significant difference (Fig. 2B). CXCL8 significantly upregulated the expression of RhoA at 12 h, but did not at 4 h. The CXCL8-upregulated Cdc42 expression at 12 h was significantly attenuated by heparinase III.

### Heparan sulfate mediates CXCL8-regulated Rac1 expression in HUVEC

The expression of Rac1 in HUVECs in the presence of CXCL8 was time-dependent (Figs. 2C and 2D). Rac1 was not significant changed by heparinase III and/or CXCL8 treatments at 4 h. Rac1 was significantly upregulated by CXCL8 at 12 h, and this was attenuated by heparinase III.

### Heparan sulfate mediates CXCL8-regulated RhoA expression in HUVEC

The expression of RhoA induced by CXCL8 was also time-dependent (Figs. 2E and 2F). At 4 h, RhoA was slightly upregulated by heparinase III with or without CXCL8 (Fig. 2E), with no significant difference (Fig. 2F). Compared with control, CXCL8 significantly induced the expression of RhoA at 12 h, but did not at 4 h. The changes at 12 h in RhoA expression induced by CXCL8 was significantly abolished by heparinase III.

### Heparan sulfate mediates CXCL8-increased Rho GTPases activity

**Figure 3:**
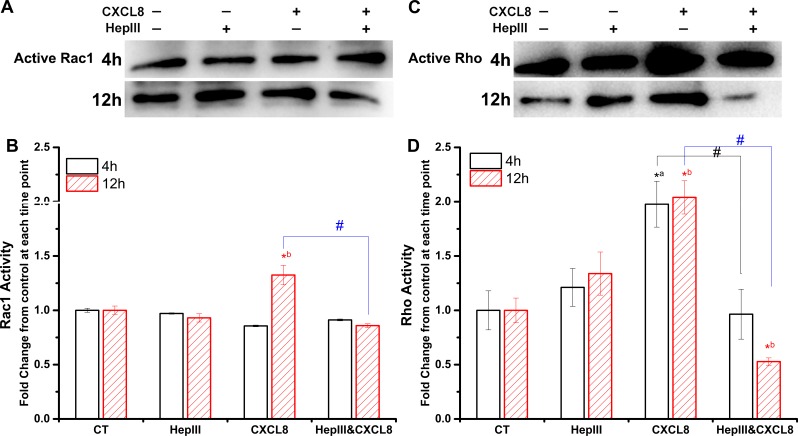
Rho GTPases activity. After treatments, the Rho GTPase activity in HUVECs was detected by using the active Rho/Rac1 pulldown assay, and quantitative data were obtained. (A and B) CXCL8 increased the Rac1 activity at 12 h, and this was abolished by heparinase III; (C and D) Heparinase III significantly reduced the CXCL8-induced Rho activity at both 4 h and 12 h. ^∗a^*P* < 0.05 vs. CT at 4 h; ^∗b^*P* < 0.05 vs. CT at 12 h; #*P* < 0.05.

We further tested the Rho GTPase activity by using the active Rho/Rac1 pull-down and detection kit. Results revealed that CXCL8 increased the Rac1 activity at 12 h, and it was abolished by heparinase III (Figs. 3A and 3B). Also, CXCL8 increased the Rho activity at both 4 h and 12 h (Figs. 3C and 3D). Heparinase III significantly reduced the CXCL8-induced Rho activity (Figs. 3C and 3D).

### Remodeling of actin cytoskeleton by CXCL8 is also heparan sulfate mediated

**Figure 4:**
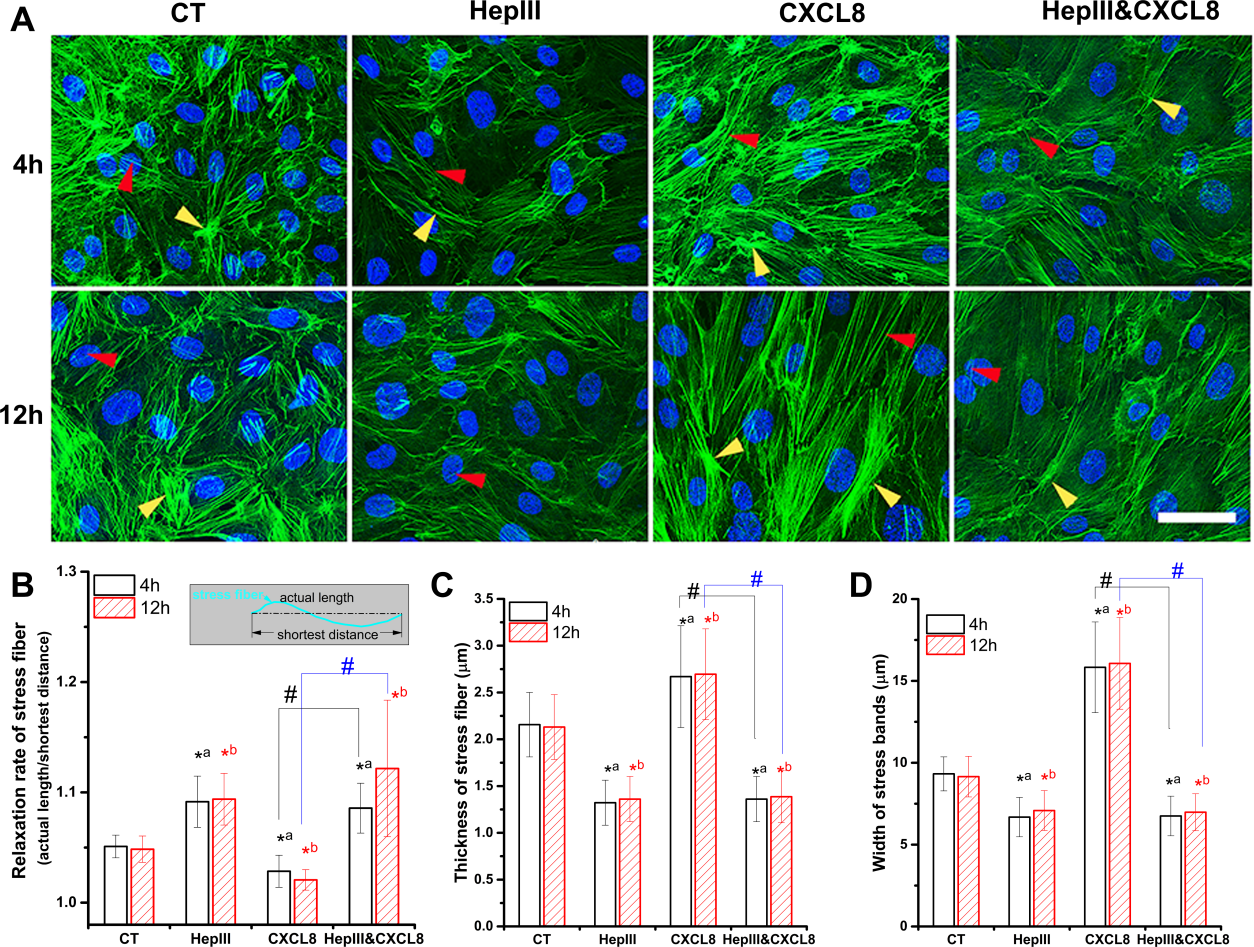
Heparinase III inhibited the CXCL8-induced actin cytoskeleton reorganization. After treatments, cells were fixed in 2% paraformaldehyde, permeabilized with 1% Triton X-100, and stained with BODIPY^®^ FL phallacidin to visualize the actin cytoskeleton (Green) by confocal microscopy (A). Blue indicates cell nuclei stained by DAPI. Red and yellow arrow heads indicate stress fibers and stress bands, respectively. Scale bar: 50 µm. (B) Relaxation rate of stress fiber, which defined as ratio of actual length of a stress fiber to shortest distance between both ends (as shown in the rectangle box). *n* = 30 stress fibers. (C) Thickness of stress fiber. *n* = 30 stress fibers. (D) Width of stress bands. *n* = 30 stress bands. ^∗a^*P* < 0.05 vs. CT at 4 h; ^∗b^*P* < 0.05 vs. CT at 12 h; #*P* < 0.05.

The changes in actin cytoskeleton in the presence of CXCL8 and/or heparinase III were investigated (Fig. 4). In control cells, the dense peripheral actin bands were present at the cell periphery of HUVECs, in which, the disordered actin filaments were organized into a loose network (Figs. 4A and 4B). In the presence of heparinase III, the stress fiber thickness (Fig. 4C) and band width (Fig. 4D) were decreased, suggesting actin network did depolymerized. CXCL8 increased stress fibers thickness (Fig. 4C) and band width (Fig. 4D), suggesting CXCL8 induced an obvious polymerization and polarization of actin cytoskeleton. The decrease in relaxation rate indicated an increased tension in HUVECs under CXCL8 exposure (Fig. 4B). Co-treatment of heparinase III with CXCL8 almost completely suppressed the remodeling of actin cytoskeleton induced by CXCL8 alone (Fig. 4), showing an important role of heparan sulfate in cytoskeleton reorganization.

## Discussion

We presently validated the crucial prerequisite of heparan sulfate for induction of wound healing and endothelial cell migration by chemokines, and mechanistically demonstrated critical roles of heparan sulfate in the CXCL8-regulated expression and activity of Rho GTPases and the reorganization of actin cytoskeleton.

It was demonstrated that GAGs interact selectively with chemokines (Kuschert et al., 1999). Based on site-directed mutagenesis, molecular modeling and NMR spectroscopy, it was demonstrated that the residues including H18, K20, R60, K64, K67 and R68 in CXCL8 participated in its interaction with heparin and heparan sulfate (Pichert et al., 2012b). GAGs involved in the cellular responses and the regulation of leukocyte recruitment (Carveth et al., 1989). Once the C-terminal GAG-binding domain on CXCL8 was deleted, it was failed to attract leukocyte with the same extent as native CXCL8 *in vitro* and *in vivo* (Middleton et al., 1997). On the other hand, soluble GAGs binding with CXCL8 to form complexes that are unable to bind the G-protein-couple chemokine receptors CXCR1 and CXCR2, inhibit the CXCL8-induced neutrophil calcium flux (Kuschert et al., 1999). Those findings indicate the binding of CXCL8 to surface endothelial GAGs is a crucial prerequisite for cell migration. In the present study, by using heparinase III to selectively cleave HS on HUVECs, the enhancement of wound healing by CXCL8 was greatly attenuated. This highlighted the biological importance of the interaction between CXCL8 and endothelial GAGs in EC migration and wound healing.

Cell migration is a complex process, which integrated the extracellular signals that initiated by chemokine or mechanical stimuli into cellular migration machinery via triggering the activation or aggregation of receptors at the cell surface and transcriptional regulation of motogenic gene products, such as Rho GTPases (Zeng et al., 2012b). Rho GTPases (including Cdc42, Rac1 and RhoA) play central roles in cell migration (Nobes & Hall, 1995). For example, Cdc42 controls the formation of protrusion and filopodia. Rac1 induces the formation of protrusion and lamellipodia. RhoA mediates the formation of stress fibers.

CXCL8 activated both the receptors CXCR1 and CXCR2 in human lung microvascular endothelial cells (HLMECs) and immortalized dermal human microvascular endothelial cells (HMECs) (Schraufstatter, Chung & Burger, 2001). Role and underlying mechanism of CXCR1/2 in CXCL8-induced EC migration have been well-investigated. It was suggested that CXCL8 initially activates RhoA and actin stress fiber formation in ECs due to activation of the CXCR1 (1–2 min), and that Rac mediated the responses of cell retraction and gap formation between neighboring cells is a function of activation of CXCR2 (15 min) in HMECs (Schraufstatter, Chung & Burger, 2001). We previously also demonstrated a quick upregulation of Rac1 expression by CXCL8 within 5 min to induce membrane ruffles via phosphoinositide 3-kinase (PI_3_ K), and a upregulation of Rac1 and RhoA expression for longer time (1–6 h) to induce the formation of stress fibers in EA.hy926 cells (Lai et al., 2011). As endothelial glycocalyx covered over the cell surface, the receptors might conceal by the GAGs. CXCL8 activates signal transduction processes leading to desensitization, internalization, and recycling of its receptors (CXCR1/CXCR2). To examine the detailed association of CXCL8 with HS, we need to design a set of experiments in the future.

After removal of the HS GAGs, we found the significant reduction of migration during 4–12 h, and the dramatically suppressed expression of Cdc42, Rac1 and RhoA at 12 h, respectively, compared with that in the presence of CXCL8. Interestingly, the wound healing/EC migration, and the reorganization of actin cytoskeleton were significantly attenuated by removal of HS, but we have not detected significant changes in Cdc42, Rac1 and RhoA expression at 4 h. The small GTPases (cdc42, Rac1, and Rho) involved in regulating the actin cytoskeleton and cell motility. Indeed, the high level of intracellular expression of small GTPases such as RhoA facilities its translocation to the membrane where it is activated, resulting in the stimulation of the RhoA-ROCK-actomyosin system, and leading to migration (Yoshioka, Nakamori & Itoh, 1999). By using the active Rho/Rac1 pull-down assay, we found that CXCL8 increased the Rac1 activity at 12 h, and it was abolished by heparinase III. Also, CXCL8 increased the Rho activity at both 4 h and 12 h, and heparinase III significantly reduced the CXCL8-induced Rho activity. It was suggested that the roles of HS in CXCL8-induced EC migration is associated with Rho GTPases.

In conclusion, there is an important biological role of the interaction between CXCL8 and endothelial GAGs in wound healing. As the chemokines induced endothelial cell migration might contribute to the wound healing after vascular injury, and to the cell recruitment in inflammation and metastatic tumor to promote angiogenesis, targeting to GAGs provides a promise strategy for therapeutic angiogenesis.

## Additional Information and Declarations

### Data Availability

The following information was supplied regarding data availability: Raw data is contained in the figures in this manuscript.

## References

[ref-1] Baggiolini M (2015). CXCL8—the first chemokine. Frontiers in Immunology.

[ref-2] Carveth HJ, Bohnsack JF, McIntyre TM, Baggiolini M, Prescott SM, Zimmerman GA (1989). Neutrophil activating factor (NAF) induces polymorphonuclear leukocyte adherence to endothelial cells and to subendothelial matrix proteins. Biochemical and Biophysical Research Communications.

[ref-3] Fu BM, Tarbell JM (2013). Mechano-sensing and transduction by endothelial surface glycocalyx: composition, structure, and function. Wiley Interdisciplinary Reviews: Systems Biology and Medicine.

[ref-4] Gales D, Clark C, Manne U, Samuel T (2013). The chemokine CXCL8 in carcinogenesis and drug response. ISRN Oncol.

[ref-5] Giantsos-Adams KM, Koo AJ, Song S, Sakai J, Sankaran J, Shin JH, Garcia-Cardena G, Dewey CF (2013). Heparan sulfate regrowth profiles under laminar shear flow following enzymatic degradation. Cellular and Molecular Bioengineering.

[ref-6] Kuschert GS, Coulin F, Power CA, Proudfoot AE, Hubbard RE, Hoogewerf AJ, Wells TN (1999). Glycosaminoglycans interact selectively with chemokines and modulate receptor binding and cellular responses. Biochemistry.

[ref-7] Lai Y, Shen Y, Liu X-H, Zhang Y, Zeng Y, Liu Y-F (2011). Interleukin-8 induces the endothelial cell migration through the activation of phosphoinositide 3-kinase-Rac1/RhoA pathway. International Journal of Biological Sciences.

[ref-8] Middleton J, Neil S, Wintle J, Clark-Lewis I, Moore H, Lam C, Auer M, Hub E, Rot A (1997). Transcytosis and surface presentation of IL-8 by venular endothelial cells. Cell.

[ref-9] Moon JJ, Matsumoto M, Patel S, Lee L, Guan JL, Li S (2005). Role of cell surface heparan sulfate proteoglycans in endothelial cell migration and mechanotransduction. Journal of Cellular Physiology.

[ref-10] Nobes CD, Hall A (1995). Rho, rac, and cdc42 GTPases regulate the assembly of multimolecular focal complexes associated with actin stress fibers, lamellipodia, and filopodia. Cell.

[ref-11] Pichert A, Samsonov SA, Theisgen S, Thomas L, Baumann L, Schiller J, Beck-Sickinger AG, Huster D, Pisabarro MT (2012a). Characterization of the interaction of interleukin-8 with hyaluronan, chondroitin sulfate, dermatan sulfate and their sulfated derivatives by spectroscopy and molecular modeling. Glycobiology.

[ref-12] Pichert A, Schlorke D, Franz S, Arnhold J (2012b). Functional aspects of the interaction between interleukin-8 and sulfated glycosaminoglycans. Biomatter.

[ref-13] Proudfoot AE (2006). The biological relevance of chemokine-proteoglycan interactions. Biochemical Society Transactions.

[ref-14] Schraufstatter IU, Chung J, Burger M (2001). IL-8 activates endothelial cell CXCR1 and CXCR2 through Rho and Rac signaling pathways. American Journal of Physiology Lung Cellular and Molecular Physiology.

[ref-15] Tarbell JM, Ebong EE (2008). The endothelial glycocalyx: a mechano-sensor and -transducer. Science Signaling.

[ref-16] Tarbell JM, Pahakis MY (2006). Mechanotransduction and the glycocalyx. Journal of Internal Medicine.

[ref-17] Tarbell JM, Simon SI, Curry FR (2014). Mechanosensing at the vascular interface. Annual Review of Biomedical Engineering.

[ref-18] Thi MM, Tarbell JM, Weinbaum S, Spray DC (2004). The role of the glycocalyx in reorganization of the actin cytoskeleton under fluid shear stress: a “bumper-car” model. Proceedings of the National Academy of Sciences of the United States of America.

[ref-19] Vicente-Manzanares M, Webb DJ, Horwitz AR (2005). Cell migration at a glance. Journal of Cell Science.

[ref-20] Weinbaum S, Tarbell JM, Damiano ER (2007). The structure and function of the endothelial glycocalyx layer. Annual Review of Biomedical Engineering.

[ref-21] Yao Y, Rabodzey A, Dewey CF (2007). Glycocalyx modulates the motility and proliferative response of vascular endothelium to fluid shear stress. AJP: Heart and Circulatory Physiology.

[ref-22] Yoshioka K, Nakamori S, Itoh K (1999). Overexpression of small GTP-binding protein RhoA promotes invasion of tumor cells. Cancer Research.

[ref-23] Zeng Y, Adamson RH, Curry FR, Tarbell JM (2014). Sphingosine-1-phosphate protects endothelial glycocalyx by inhibiting syndecan-1 shedding. AJP: Heart and Circulatory Physiology.

[ref-24] Zeng Y, Ebong EE, Fu BM, Tarbell JM (2012a). The structural stability of the endothelial glycocalyx after enzymatic removal of glycosaminoglycans. PLoS ONE.

[ref-25] Zeng Y, Shen Y, Huang XL, Liu XJ, Liu XH (2012b). Roles of mechanical force and CXCR1/CXCR2 in shear-stress-induced endothelial cell migration. European Biophysics Journal.

[ref-26] Zeng Y, Sun HR, Yu C, Lai Y, Liu XJ, Wu J, Chen HQ, Liu XH (2011). CXCR1 and CXCR2 are novel mechano-sensors mediating laminar shear stress-induced endothelial cell migration. Cytokine.

[ref-27] Zeng Y, Tarbell JM (2014). The adaptive remodeling of endothelial glycocalyx in response to fluid shear stress. PLoS ONE.

[ref-28] Zeng Y, Waters M, Andrews A, Honarmandi P, Ebong EE, Rizzo V, Tarbell JM (2013). Fluid shear stress induces the clustering of heparan sulfate via mobility of glypican-1 in lipid rafts. AJP: Heart and Circulatory Physiology.

